# *Sphingobacterium spiritivorum* bacteremia due to cellulitis in an elderly man with chronic obstructive pulmonary disease and congestive heart failure: a case report

**DOI:** 10.1186/s13256-017-1445-6

**Published:** 2017-09-30

**Authors:** Arata Hibi, Yuka Kumano

**Affiliations:** 1 Division of Nephrology and Rheumatology, Department of Internal Medicine, Kariya Toyota General Hospital, 5-15, Sumiyoshi-cho, Kariya, Aichi 448-8505 Japan; 2 Department of Dermatology, Kariya Toyota General Hospital, 5-15, Sumiyoshi-cho, Kariya, Aichi 448-8505 Japan

**Keywords:** *Sphingobacterium spiritivorum*, Cellulitis, Bacteremia

## Abstract

**Background:**

*Sphingobacterium spiritivorum* is a glucose non-fermenting Gram-negative rod, formerly classified as one of the *Flavobacterium* species. It is characterized by a large number of cellular membrane sphingophospholipids. *Sphingobacterium* species are ubiquitous and isolated from natural environments, such as soil and water. However, they rarely cause infections in humans. Only a limited number of cases have been reported in elderly and immunocompromised patients with underlying diseases and predisposing factors.

**Case presentation:**

An 80-year-old Japanese man with chronic obstructive pulmonary disease and congestive heart failure visited the Kariya Toyota General Hospital, Aichi, Japan with the chief complaint of fever accompanied by chills and left leg pain. At initial presentation, he was distressed and dyspneic. He was febrile (38.8 °C), and his left foot was swollen with reddening and tenderness. We diagnosed him as having cellulitis, and he was hospitalized for antibiotic therapy. Initially, he was treated with intravenously administered cefazolin, but after the isolation of a glucose non-fermenting Gram-negative rod from blood cultures, we decided to switch cefazolin to intravenously administered meropenem on day 4, considering the antibiotic resistance of the causative organism. The causative organism was identified as *S. spiritivorum* on day 6. His condition gradually stabilized after admission. Meropenem was switched to orally administered levofloxacin on day 12. He was discharged on day 16 and treated successfully without any complications.

**Conclusions:**

Although *S. spiritivorum* is rare, with limited cases isolated from cellulitis, it should be considered as a causative organism in elderly and immunocompromised patients with cellulitis. Blood cultures are the key to correct diagnosis and appropriate treatment.

## Background

*Sphingobacterium spiritivorum* (*S. spiritivorum*) is a glucose non-fermenting Gram-negative rod (GNF-GNR), formerly classified as one of the *Flavobacterium* species [1]. It is characterized by a large number of cellular membrane sphingophospholipids [1]. *Sphingobacterium* species are ubiquitous and isolated from natural environments, such as soil and water. However, they rarely cause infection in humans. Only a limited number of cases have been reported in elderly and immunocompromised patients with underlying disease and predisposing factors [2–6]. However, *S. spiritivorum* has the potential of causing fatal infections and bacteremia, particularly in elderly and immunocompromised patients. Although our case is not the first case report of *S. spiritivorum* infection isolated from humans, we propose that it is important to consider *S. spiritivorum* as a causative organism in selected patients with cellulitis.

## Case presentation

An 80-year-old Japanese man presented to our hospital with complaints of fever and left leg pain, as well as bilateral lower extremity swelling. Although mild edema was always observed in his bilateral lower extremities, it gradually worsened 1 week prior to admission. On the day of admission, he had high fever accompanied by chills. He denied any recent leg trauma. His past medical history was significant for pulmonary tuberculosis at 30 years of age, chronic obstructive pulmonary disease (COPD), and congestive heart failure (CHF). A pacemaker was inserted because of atrial fibrillation (AF) with symptomatic bradycardia. He was an ex-tobacco smoker (100 pack year history) and did not drink alcohol. His maintenance medications were dabigatran (220 mg), furosemide (20 mg), and an inhaled corticosteroid/long-acting β_2_-agonist.

**Fig. 1:**
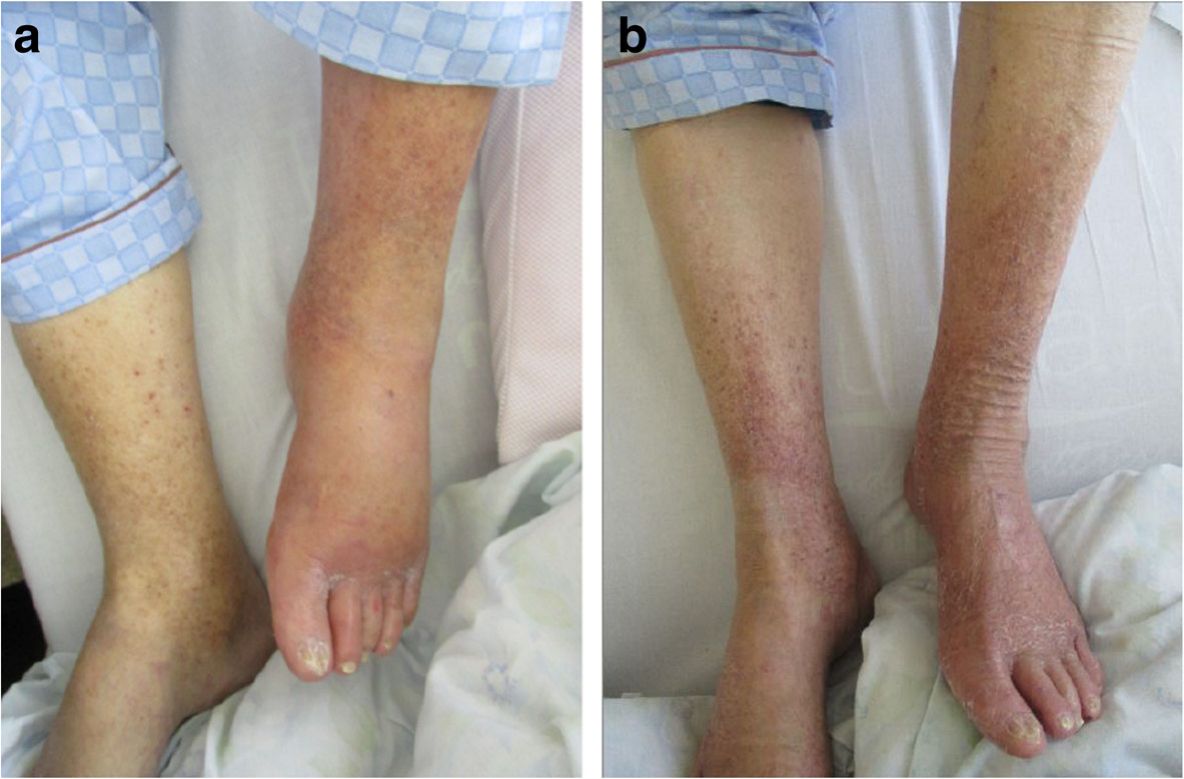
Appearance of lower extremities of the patient on the day of admission (**a**) and after treatment (**b**). On the day of admission, reddening and swelling were observed on the left leg. Dermatophyte was positive in the toe webs as per a potassium hydroxide test

At initial presentation, he was alert and oriented but appeared distressed. His vital signs were as follows: body temperature, 38.8 °C; blood pressure, 135/90 mmHg; heart rate, 96 beats per minute with irregular rhythm; blood oxygen saturation, 93% with room air; and respiratory rate, 22 breaths per minute. He had a barrel-shaped chest, coarse crackles were heard over the lung base, and wheezes were heard over both lung fields. Pitting edema was observed in his lower extremities. His left foot had more erythema and edema than his right foot (Fig. 1). His left lower extremity was warm and tender to touch. There were no skin breaks or other potential infection entry sites. Tinea pedis was ascertained by a potassium hydroxide test.

Arterial blood gas analysis showed the following results: pH, 7.408; partial pressure of carbon dioxide, 44 mmHg; partial pressure of oxygen, 72 mmHg; bicarbonate, 22.5 mmol/L; and lactate, 1.8 mmol/L. A complete blood count revealed the following results: white blood cells, 9,000/μL; red blood cells, 384 × 10^4^/μL; hemoglobin, 12.4 g/dL; and platelets, 9.6 × 10^4^/μL. Chemistry results were as follows: serum creatinine, 0.81 mg/dL; blood urea nitrogen, 19.0 mg/dL; albumin, 3.7 g/dL; total bilirubin, 2.1 mg/dL; aspartate aminotransferase, 43 U/L; alanine aminotransferase, 22 U/L; C-reactive protein, 0.36 mg/dl; and brain natriuretic peptide (BNP), 471 pg/mL (our patient’s basal BNP level was approximately 100 pg/mL). A coagulation test showed prolonged prothrombin time-international normalized ratio (1.29) and activated partial thromboplastin time (52.3 seconds). A chest X-ray showed a nodular lesion on the right pulmonary apex compatible with previous tuberculous infection without any infiltrations. An electrocardiogram showed AF with pacemaker rhythm without any sensing and pacing failures. Based on these results, we diagnosed our patient as having cellulitis and immediately admitted him to hospital because his respiratory status continued to worsen, necessitating supplemental oxygen therapy for dyspnea relief. We also considered a risk of sepsis and mortality because his sequential organ failure assessment score was 5 points (2 points greater than baseline) at initial presentation [7].

**Fig. 2:**
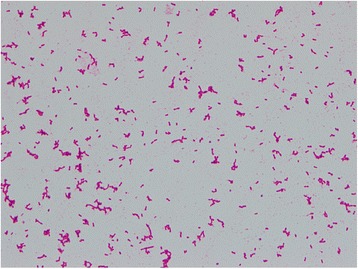
Gram stain of the organism isolated from blood culture (magnification, ×1000). Gram-negative short rods were seen

Cefazolin (1 g every 8 hours) was initially administered intravenously to treat his cellulitis, but after 25 hours of incubation, two sets of aerobic blood culture bottles (BD BACTEC™ Plus Aerobic/F Medium; BD Diagnostics, Sparks, MD, USA) were found to be positive (detected by BD BACTEC™ FX, Blood Culture System; BD Diagnostics, Sparks, MD, USA) for GNRs (Fig. 2). The positive culture broth was inoculated onto a blood agar plate (BD BBL™ Trypticase™ Soy Agar with 5% Sheep Blood; Nippon Becton Dickinson Company, Fukushima, Japan) and light yellow colonies were observed after incubation of 24 hours at 37 °C. The causative organism was confirmed as GNF-GNR on day 4. Accordingly, we switched antibiotics to intravenously administered meropenem (1 g every 8 hours) on the same day, considering antibiotic resistance. On day 6, the causative organism was identified as *S. spiritivorum*. It was identified by BD PHOENIX™ System (BD Diagnostics, Sparks, MD, USA) and matrix-assisted laser desorption/ionization time of flight mass spectrometry, using Microflex LT with MALDI Biotyper version 3.1 database (Bruker Daltonik, Bremen, Germany). Our patient’s condition gradually improved with the antibiotic use. We decided to switch antibiotics to orally administered levofloxacin (500 mg/day) on day 12, considering the antibiotic sensitivity of *S. spiritivorum* isolated from the blood culture (Table 1). Trimethoprim/sulfamethoxazole was an alternative but was not used out of concern for adverse drug reactions considering our patient’s age. He was discharged on day 16 without any complications, and the antibiotic was discontinued on the same day. We followed up with him 2 weeks after discharge, during which he did not have any residual symptoms related to cellulitis.

**Table 1:** Antibiotics sensitivity of *Sphingobacterium spiritivorum* isolated from the present case

Antibiotics	Minimal inhibitory concentration (μg/mL)	Sensitivity
Piperacillin	16	S
Cefoperazone	32	I
Ceftazidime	≤4	S
Cefepime	4	S
Latamoxef	>32	R
Imipenem/cilastatin	≤1	S
Meropenem	≤1	S
Piperacillin/tazobactam	8	S
Aztreonam	>16	R
Amikacin	>32	R
Tobramycin	>8	R
Gentamycin	>8	R
Minocycline	≤1	S
Trimethoprim/sulfamethoxazole	≤20	S
Levofloxacin	≤1	S
Ciprofloxacin	≤0.5	S

*I* intermediate, *R* resistant, *S* susceptible

## Discussion

*Sphingobacterium* species are aerobic, Gram-negative, short rod, non-motile, non-spore-forming bacteria. They are oxidase-positive, catalase-positive, and urease-positive and indole-negative and produce light yellow colonies on blood agar plates [1]. Thus far, more than 20 species in the genus *Sphingobacterium* have been reported based on 16S ribosomal ribonucleic acid gene sequencing [8] and the number of isolated species is increasing. *S. spiritivorum* was first isolated from a human clinical specimen by Holmes *et al*. in 1982 [9] and was initially described as *Flavobacterium spiritivorum*. In 1983, Yabuuchi *et al*. first proposed *Sphingobacterium* as a new genus [10]. The genus *Sphingobacterium* differs from the genus *Flavobacterium* by high cellular membrane concentrations of sphingophospholipid and ceramide. Naka *et al*. performed a structural analysis of sphingophospholipids in *S. spiritivorum*, thereby purifying a novel sphingolipid among eukaryotic and prokaryotic cells [11].

*Sphingobacterium* species are ubiquitous and commonly isolated from soil, plants, and water, but rarely from human infection sites. *Sphingobacterium multivorum* and *S. spiritivorum* were isolated from very few existing cases. Lambiase *et al*. reported the isolation of *S. multivorum* and *S. spiritivorum* from the sputum of patients with cystic fibrosis [12]. Recently, the first human case of *Sphingobacterium hotanense* infection in an elderly patient was reported [13]. In that case, scratches on the right arm caused by a rooster were the suspected infection entry site from soil.

*Sphingobacterium* species are resistant to commonly used antibiotics [1]. *S. multivorum* can produce an extended-spectrum β-lactamase and a metallo-β-lactamase, which make it resistant to third-generation cephalosporins and carbapenems, respectively [14]. *S. spiritivorum* is susceptible to carbapenems. Quinolones, trimethoprim-sulfamethoxazole, and ceftazidime are effective *in vitro,* which is compatible with previous clinical reports [12]. *S. spiritivorum* isolated from the present case was susceptible to the antibiotics listed above. In the present case, we observed a good clinical course with intravenously administered meropenem followed by orally administered levofloxacin.

We identified five previously reported cases of *S. spiritivorum* infection in the English literature [2–6] (Table 2). Three cases were caused by cellulitis [2, 3, 6] and two cases by catheter-related blood stream infection [4, 5]. In most of these cases, the patients had predisposing factors and underlying diseases, such as Parkinson’s disease (with chronic venous stasis due to akinesia and injuries from frequent falls, which are risk factors for cellulitis) [2, 3], refractory anemia [4], acute myeloid leukemia treated with chemotherapy [5], and end-stage renal disease on hemodialysis [6]. One case of extrinsic allergic alveolitis (hypersensitivity pneumonitis) caused by *S. spiritivorum* [15] was not included because it was not a direct infection but was caused by a hypersensitivity reaction against organism-derived allergens [16]. In our case, edema due to CHF was a risk factor for cellulitis [17]. Aging and COPD can also increase susceptibility to infections [18, 19]. Tinea pedis is a risk factor for cellulitis [20] because it may provide entry sites for infections [21] and changes in bacterial flora [22].

**Table 2:** Previously reported five cases of *Sphingobacterium spiritivorum* infections and the present case

Case number and Reference	Reported year	Age/Sex	Underlying diseases and predisposing factors	Source of isolation	Diagnosis	Antibiotics	Clinical outcome
Case 1 [2]	2002	72/M	Parkinson’s disease	Blood	Cellulitis	Cefazolin followed by ampicillin/sulbactam	Complete recovery
Case 2 [3]	2003	84/M	Refractory anemia	Blood	Cellulitis	Amoxicillin/clavulanate	Complete recovery
Case 3 [4]	2013	68/F	Acute myeloid leukemia treated with chemotherapy	Blood	CRBSI	Cefepime followed by ciprofloxacin	Died
Case 4 [5]	2016	80/F	ESRD on hemodialysis via tunneled central venous dialysis catheter; DM	Blood	CRBSI	Trimethoprim followed by meropenem and ciprofloxacin	Complete recovery
Case 5 [6]	2016	89/M	Parkinson’s disease; skin tears and abrasion due to multiple falls	Blood	Cellulitis	Piperacillin/tazobactam followed by amoxicillin/clavulanate	Complete recovery
The present case	2017	80/M	COPD; edema due to CHF; tinea pedis	Blood	Cellulitis	Meropenem followed by levofloxacin	Complete recovery

*CHF* congestive heart failure, *COPD* chronic obstructive pulmonary disease, *CRBSI* catheter-related blood stream infection, *DM* diabetes mellitus, *ESRD* end-stage renal disease, *F* female, *M* male

Although obtaining blood cultures of patients with cellulitis may not be cost effective, given the low rate of positive blood cultures (2.0%) [23], we could not have made a correct diagnosis in the present case without blood cultures. Mills and Chen reviewed several studies and concluded that obtaining blood cultures does not significantly alter treatment or aid in diagnosing the causative organism in immunocompetent patients with acute cellulitis [24]. In addition, the current Infectious Diseases Society of America (IDSA) guidelines do not recommend routine performance of blood cultures in patients with cellulitis; however, performing blood cultures is recommended in patients with malignancy, chemotherapy, neutropenia, severe cell-mediated immunodeficiency, immersion injuries, and animal bites [25]. Peralta *et al*. reported the absence of previous antibiotic treatment and the presence of two or more comorbid factors including obesity, COPD, diabetes, alcohol addiction, liver cirrhosis, CHF, and immunocompromised condition were associated with bacteremia in patients with cellulitis [26]. Lee *et al*. proposed an initial diagnostic prediction model with four independent predictors for estimating probability of bacteremia in patients with cellulitis: age ≥ 65 years, involvement of non-lower extremities, liver cirrhosis, and systemic inflammatory response syndrome [27]. In a recent study, van Daalen *et al*. reported the blood culture positivity rate was higher than the rates reported by IDSA guidelines in hospitalized patients with skin and soft tissue infections, particularly in patients with severe comorbidity [28]. Evaluation of patients’ comorbidity is critical to making decisions to perform blood cultures in patients with cellulitis. Considering *S. spiritivorum* was isolated from blood cultures in all of the previous reports, performing blood cultures in patients with cellulitis with comorbid risk factors can be useful to identify the causative organism and important for appropriate treatment.

## Conclusions

*S. spiritivorum* is a rare causative organism of cellulitis, with a limited number of reported cases in the literature. In the present case, aging and COPD could have been the risk factors for infection, and edema due to CHF was a predisposing factor for cellulitis. Tinea pedis could have produced an infection entry site. Although our patient was initially septic, he was successfully treated by administration of targeted antibiotics. Blood cultures were key to identifying the causative organism in the present case. We should consider *S. spiritivorum* as a potential causative organism of cellulitis, particularly in patients with comorbid risk factors.

## Abbreviations

AF: Atrial fibrillation
BNP: Brain natriuretic peptide
CHF: Congestive heart failure
COPD: Chronic obstructive pulmonary disease
GNF-GNR: Glucose non-fermenting Gram-negative rod
IDSA: Infectious Diseases Society of America
